# Approximate sparse spectral clustering based on local information maintenance for hyperspectral image classification

**DOI:** 10.1371/journal.pone.0202161

**Published:** 2018-08-17

**Authors:** Qing Yan, Yun Ding, Jing-Jing Zhang, Li-Na Xun, Chun-Hou Zheng

**Affiliations:** 1 College of Electrical Engineering and Automation, Anhui University, Hefei, Anhui, China; 2 College of Computer Science and Technology, Anhui University, Hefei, Anhui, China; Universita degli Studi di Trento, ITALY

## Abstract

Sparse spectral clustering (SSC) has become one of the most popular clustering approaches in recent years. However, its high computational complexity prevents its application to large-scale datasets such as hyperspectral images (HSIs). In this paper, we propose two efficient approximate sparse spectral clustering methods for HSIs clustering in which clustering performance is improved by utilizing local information among the data. Firstly, we construct a smaller representative dataset on which sparse spectral clustering is performed. Then the labels of ground object are extending to whole dataset based on the local information according to two extending strategies. The first one is that the local interpolation is utilized to improve the extension of the clustering result. The other one is that the label extension is turned to a problem of subspace embedding, and is fulfilled by locally linear embedding (LLE). Several experiments on HSIs demonstrated that the proposed algorithms are effective for HSIs clustering.

## Introduction

Hyperspectral (HS) remote sensors can capture images in hundreds of spectral bands which provide useful information for discriminating different materials of interest in a scene. With the rapid development of imaging spectroscopy technologies, current sensors are able to acquire hyperspectral image(HSI) data with high spatial and spectral resolutions simultaneously [1]. Although abundant space-spectrum information is beneficial to improve the ability of object recognition, but on the other hand also brought some difficulties, for the existence of the Hughes phenomenon [2]. Realizing the object recognition must rely on the classification algorithm, which include two categories, i.e., the supervised approach and the unsupervised approach. Supervised techniques require the availability of a training set for training the classifier. Unsupervised methods, also known as clustering methods, perform recognition just by exploiting information conveyed by the data, without requiring any training sample set. Usually, the supervised methods offer the higher classification accuracy compared to the unsupervised ones. But in some circumstances, such as hyperspectral image recognition problem, it is necessary to resort to unsupervised techniques because training information acquisition is difficult and expensive [3]. As is well known to us all, HSIs are typical high-dimensional data with large spectral variability, high dimensionality, and complex structures, which makes HSI clustering to be a very challenging task [4].

Nowadays, many different clustering methods for HSIs have been proposed. The existing clustering algorithms for HSIs can be coarsely divided into the following four categories, i.e., Iterative methods, statistical methods, algebraic approaches and spectral clustering-based methods. The Iterative-based clustering methods, such as k-means [5], FCM [6] and K-flats [7], alternate between assigning points to subspaces and fitting a subspace to each cluster. Statistical approaches, such as Mixtures of Probabilistic PCA (MP-Principal Component Analysis) [8] and Multi-Stage Learning (MSL) [9], assume that the distribution of the data inside each subspace is Gaussian and alternate between data clustering and subspace estimation by applying Expectation Maximization (EM) to a mixture of probabilistic PCAs. But statistical approaches are sensitive to initialization and need to know the number of the subspaces. Algebraic approaches such as generalized principal component analysis (GPCA) [10] fit the data with a polynomial, whose gradient at a point gives the normal vector to the subspace containing that point. However, the flaw of GPCA is that it is sensitive to noise and outliers.

Spectral clustering-based methods have two main categories, local spectral clustering-based approaches and globe spectral clustering-based approaches. Local subspace affinity (LSA) [11], locally linear manifold clustering [12], etc., which belong to the first category, uses local information to build a similarity between pairs of points. But these methods have unsatisfied effect when the points located at the intersection of two subspaces and have difficulties in how to choice the neighborhood size. The reason is that the categorical attribute of neighborhood can be hardly decided. The global spectral clustering-based approaches, such as spectral curvature clustering (SCC) [13], try to resolve this problem. These methods build similarities based on not local but global information. However, they need to obtain some a priori information, such as the number and dimensions of the subspaces, and assume that all the subspaces have the same dimensions.

In recent years, Sparse Subspace Clustering (SSC) is becoming a newly developed spectral clustering-based framework for data clustering [14, 15].Sparse subspace clustering pursues a sparse representation of high-dimensional data and uses it to build the affinity matrix. The clustering result is finally obtained by means of spectral clustering (SC). The key to sparse subspace clustering is to design a good representation model which can reveal the real subspace structure of high-dimensional data [16]. Sparse subspace clustering has been successfully applied to different research fields, such as face clustering [17], motion segmentation [18], and so on. For HSIs, pixels coming from the same land-cover, which may have similar spectra characteristics, have a high probability that they lie in the same subspace [19]. So, each kind of land-cover material can be grouped into a subspace. Therefore, subspace theory can be used to model the clustering of HSIs. Sun et al. [20] proposed an improved sparse subspace clustering (ISSC) method to select an appropriate band subset for hyperspectral imagery (HSI) classification. The angular similarity measurement is presented and utilized to construct the similarity matrix. Zhang et al. proposed a novel spectral–spatial sparse subspace clustering (S^4^C) algorithm for hyperspectral remote sensing images clustering [19]. Considering the spectral and spatial properties of HSIs, the high spectral correlation and rich spatial information of the HSIs are taken into consideration in the SSC model to obtain a more accurate coefficient matrix. However, directly applying the SSC algorithm to HSIs usually is difficult for the huge calculated amount. The order of magnitude of the HSIs often reaches to several hundred thousand and more, which cannot be afforded by normal computer. The conventional researches [19] on HSIs often cut the original image into small piece.

Aimed at this problem, several methods have been developed to speed up the spectral clustering algorithms, which can be loosely classified into two types. One type accelerates spectral clustering by reducing the computation of the eigen-decomposition of the Laplacian graph, such as the Nyström method [21]. This kind of method has the drawback of complex calculation. The other type of the approximate spectral clustering methods samples a representative data set on which the spectral clustering is performed, and the result is extended to the whole data set. Under this framework, one is based on k-means clustering (KASP) [22] and the other is based on random projection trees (RASP) [23]. KASP has obviously advantage than Nyström method, no matter from accuracy, calculated amount and memory requirements. But in this kind of method, if the label of representative data is falsely clustered, all related points will be wrongly assigned. Moreover, because of the quantification of k-means, it is possible that some data points which are close together may be assigned to different clusters. Cao et al. proposed an improved approximate spectral clustering method based on local information (LI-ASP) [24]. In this article, the local interpolation is adopted to improve the extension of the clustering result on the small representative set.

Inspired by the aforementioned works, in this paper, we propose two approximate sparse subspace clustering methods. Firstly, a smaller representative dataset is constructed, and the sparse spectral clustering (SSC) is performed on this small dataset. Then the labels obtained by SSC are extended to whole dataset based on local information. We design two methods for labels extending. One method is that the whole data is interpolated into a new space based on the local relationship with representative dataset. The other method is that the label extension is turned to a problem of subspace embedding. So the whole data will be embedded to the space which is spanned by representative dataset, and the embedding is fulfilled by locally linear embedding (LLE). From these two ways of extension, we can get a new space representation included all data with different pattern. Finally, in this new space, the k-means is performed on all data, and the final clustering result is obtained. These ways of extension can be supervised by data local information, so the more precise clustering result can be gained. The contributions of this paper are summarized as follows. First, to the best of our knowledge, we are the first ones to fulfill the clustering for HSIs on the point of pixels with the approximate SSC(ASSC) algorithm. Second, Introducing the ASSC based on local interpolation (LI-ASSC) to SSC, and the procedure of extending the label is supervised by local spectral features. Third, based on Local Linear Embedding (LLE), a novel extending rule is proposed (LLE-ASSC), which can effectively keep the local linear relationship.

The remainder of this paper is organized as follows. Section II briefly introduces the SSC model for HSIs and the approximate spectral clustering based on Local information-based (LI-ASP). In Section III, we propose the LI-ASSC algorithm for HSIs clustering, which is focus on approximate SSC for hyperspectral image clustering with local interpolation. And the ASSC based on LLE (LLE-ASSC) is also introduced in this section, which emphasis is to fulfill approximate SSC by subspace embedding. The experimental results are given in Section IV. Section V concludes this paper and outlines future works.

## HSI clustering via the SSC model

### HSI clustering via the SSC model

In this section, the HSI clustering scheme with the SSC model is introduced. The HSI data can be denoted as $Z^{/}\in R^{M\times N\times D}$ which is a 3-D data cube, where *M* represents the width of the HSI image, *N* stands for the height of the image and *D* is the number of the spectral band. Before clustering, we must reorder Z^/^ into a 2-D matrix, which is denoted by $Y=\left[Z_{1}^{\prime },Z_{2}^{\prime },\ldots .Z_{MN}^{\prime }\right]$, $Y\in R^{MN\times D}$. Then, with the hyperspectral data itself being used as the dictionary, the SSC model utilizes the self-expressiveness property of the data to build the sparse representation model as follows:

$${min}_{c,\;E}\|C{\|}_{0}+\rho {\left\|E\right\|}_{F}^{2}$$

$$s.t.\;Y=YC+E,\;diag(C)=0,C^{T}1=1$$ (1)

where $C≜[c_{1},c_{2},\ldots .c_{MN}]\in R^{MN\times MN}$ is the matrix whose *i*th column corresponds to the sparse representation of Y_*i*_, **E** is a noise matrix, and parameter *ρ* balance the two terms in the objective function. The diag(**C**) = 0 is used to eliminate the trivial solution of writing a point as an affine combination of itself. In addition, the constraint **C**^*T*^ 1 = 1 ensures that it is a case of an affine subspace [14, 15].

Unfortunately, (1) is a nonconvex optimization problem, so there is no unique and stable solution. We can obtain a tractable convex optimization problem by relaxing (1) and replacing the 0-norm with the 1-norm, which yields the following convex surrogate [19]:

$${min}_{c,\;E}\|C{\|}_{1}+\rho {\left\|E\right\|}_{F}^{2}$$

$$s.t.\;Y=YC+E,\;diag(C)=0,C^{T}1=1$$ (2)

The optimization problem in (2) can be solved by the alternating direction method of multipliers(ADMM).

Next, the obtained sparse coefficient matrix **C** can be adopted to construct the adjacent matrix *w*_*ij*_ ∈**W**, which defines the weight on the edge between the data nodes in the following way:

$$w_{ij}=|c_{ij}|+|c_{ji}|$$ (3)

Algorithm1: sparse subspace clustering for HSIs

**Input**: HSI data points ${\left\{y_{i}\right\}}_{i=1}^{MN}$, which come from a union of *l* affine subspaces ${\left\{S_{i}\right\}}_{i=1}^{l}$, *l* is the parameter denoting the cluster number.

Step 1. Calculate the sparse coefficient matrix **C** of data points ${\left\{y_{i}\right\}}_{i=1}^{MN}$ using the sparse subspace clustering model (2).

Step 2. Normalize the columns of **C**as ${c_{i}\leftarrow \frac{c_{i}}{\left\|c_{i}\right\|}}_{\infty }.$

Step 3. Establish similar weighted graph W according to the sparse coefficient matrix with (3).

Step 4. Perform spectral clustering on the similarity graph.

**Output:** A 2-D matrix which records the labels of the clustering result of the HSI.

Directly applying the SSC algorithm to HSIs is usually invalid since the great computational complexity can’t be afforded by normal computer or the computational time is too long. In fact, SC has the same difficulties when applying to large-scale datasets. So many improving methods have been proposed as mentioned in Section I. In next sub-section, we will emphasis on one of them, called LI-ASP.

### Local information-based approximate spectral clustering (LI-ASP)

Cao et al. [24] thought the process of extending must reflect the relationship between whole data and representation dataset. And by analyzing, they found the local relationship is very important to extension. So a local interpolation rule is proposed for approximate spectral clustering (LI-ASP), in order to improve the extension from the representative points clustering result to the whole result. This interpolation rule is based on the assumption that the nearby points are likely to have the same labels. Because the local information is used to supervise the extension process, so this extension will not destroy the local data relationship, and can get more precise clustering result. The process of LI-ASP can be summarized as follows.

For data set **Y** = [*y*_1_,*y*_2_,…*y*_*mn*_], denoted **X** = [*x*_1_,*x*_2_,…*x*_*p*_] as the *p* randomly sampled representative points. Firstly, calculating the new representations of **X**, i.e. $X^{\prime }=\left[x_{1}^{\prime },x_{2}^{\prime },\ldots x_{p}^{\prime }\right]$., using the SC algorithm, which is just formed by the top p eigenvectors of the Laplacian graph. Then, computing the pairwise distances between original data **Y** and sampled data **X** by using (4)

$$y_{i}^{\prime }=\sum_{j\in N(i)}\exp\left(\frac{-d(x_{j},y_{i})}{\sigma^{2}}\right)x_{j}^{\prime }.$$ (4)

Where N(i) is the neighborhood set of p representative points away from the original data. Finally, performing k-means on $Y^{\prime }=\left[y_{1}^{\prime },y_{2}^{\prime },\ldots y_{n}^{\prime }\right]$, to obtain the whole labels of the dataset Y.

## HSI clustering via the approximate SSC(ASSC) model

LI-ASP is an efficient approximate SC algorithm based on local information. It focuses on improving the results of spectral clustering on the representative set and extending that result to all data. So this method significantly improves approximate SC, while still maintaining scalability to large-scale datasets. In this section, inspiring by the idea of approximate SC, we propose two approximate SSC algorithms for hyperspectral clustering, named LI-ASSC and LLE-ASSC.

### Local information based approximate SSC(LI-ASSC)

LI-ASSC is based on LI-ASP directly. The only difference is that we get the eigenvectors of representative data by SSC but not SC. Theoretically these two algorithms have identical advantages. Because of utilizing the local spectral information of HSIs, the complicated computation can be overcome, and meantime the accuracy of label extending is promoted. The proposed LI-ASSC algorithm is summarized in Algorithm 2.

Algorithm 2: *Local information based approximate SSC(LI-ASSC)* for HSIs

**Input**: HSI data points ${\left\{y_{i}\right\}}_{i=1}^{MN}$, which come from a union of *l* affine subspaces ${\left\{S_{i}\right\}}_{i=1}^{l}$, *l* is the parameter denoting the cluster number.

Step 1. Calculate the sparse coefficient matrix **C** of data points ${\left\{y_{i}\right\}}_{i=1}^{MN}$ using the sparse subspace clustering model (2).

Step 2. Normalize the columns of **C** as ${c_{i}\leftarrow \frac{c_{i}}{\left\|c_{i}\right\|}}_{\infty }$.

Step 3. Establish similar weighted graph W according to the sparse coefficient matrix with (3).

Step 4. Apply SC to the similarity graph and get the top p eigenvector, denoted by $X^{\prime }=[x_{1}^{\prime },x_{2}^{\prime },\ldots x_{p}^{\prime }].$

Step 5. Embedding **Y** to feature space based on **X**′with (4), get the new representation **Y**′.

Step 6. Perform k-means on **Y**′ to obtain the whole labels.

**Output:** A 2-D matrix which records the labels of the clustering result of the HSI.

### Local linear embedding based approximate SSC (LLE-ASSC)

In LI-ASSC, the local information of data is used to help the process of label extending keeping the local data relationship unchanged, so the clustering result should be improved. This kind of interpolation method looks the local data relationship as linear. But for actual circumstances, especially HSIs, the relationship of data has obviously nonlinear trait. So, linear interpolation cannot reflect the real local spectral characteristics. By analyzing the process of interpolation, we find that the step of extending labels can be looked upon a course of data embedding actually, i.e., a new feature space is established according to the eigenvector of representative data. Then based on the local relationship of original data with the representative set, the whole original data can be embedded to this feature space. And the whole labels can be obtained by using k-means on this new feature space.

Since HSIs have nonlinear relationship, so we must select a nonlinear analytical method to depict the data relationship. In recent years, manifold learning is one of the outstanding algorithms for nonlinear analysis. And the local linear embedding (LLE) is a famous one among the manifold learning. LLE has global optimal analytical solution, and fulfills the embedding by solving sparse matrix eigenvector, no needing iteration. So the complexity of LLE is relatively small in manifold learning and fits the big scale data, such as HSIs. So here, we use LLE to accomplish the label extending in ASSC. We named it LLE-ASSC. The details of it are summarized as following.

For every data in **X** = [*x*_1_,*x*_2_,…..,*x_p_*], $X\in R^{D\times P}$. Calculating the reconstruction weight *w*_*ij*_ by minimizing (5), and reconstructing representative data *x*_*i*_ by its *k* nearest neighbors

$$\varepsilon (W)={\sum_{i=1}^{p}\left|x_{i}-\sum_{j=1}^{k}w_{ij}x_{j}\right|}^{2}$$ (5)

Where *ε*(*W*) is the reconstruction cost function, and weight *w*_*ij*_ meets the constraint condition ∑_*j*_*w*_*ij*_ = 1 and *w*_*ij*_ = 0, if *x*_*i*_ is not a neighbor of *x*_*j*_. Then the low-dimensional vector X' is constructed by keeping *w*_*ij*_ unchanged and minimizing the following error function (6):

$$\phi (X^{'})=\sum_{i=1}^{n}{\left\|x_{i}^{'}-\sum_{X_{j}\in \Omega (X_{i})}w_{ij}x_{j}^{'}\right\|}^{2}={\left\|X^{'}(I-W^{T})\right\|}^{2}=tr[X^{'}N(X^{'}){}^{T}]$$ (6)

Where $x_{i}^{'}\in R^{d\times p}(d<D)$, $\sum_{i}x_{i}^{'}=0,and\frac{1}{n}{\sum_{i}x_{i}^{'}(x_{i}^{'})}^{T}=I$, Now, we get a new low dimensional representation **X**' of **X**.

Next, we embed each data in **Y** to this low dimensional space. For each data in **Y**, we find their *k* nearest neighbors from **X**. And like (5), the weight is got using (7)

$$\varepsilon (U)={\sum_{i=1}^{M\times N}\left|y_{i}-\sum_{j=1}^{k}u_{ij}x_{j}\right|}^{2}$$ (7)

Keeping *u*_*ij*_ unchanged, we can get the low dimensional representation of **Y** with (8), and the whole original HSI data can be embedded to this low dimensional space.

$$y_{j}^{'}=\sum_{i=1}^{k}u_{ij}^{}x_{i}^{'}$$ (8)

Finally, by performing k-means, the label result is obtained. We summarized this method in Algorithm 3.

Algorithm 3: *Local linear embedding approximate SSC(LLE-ASSC)* for HSIs

**Input:** HSI data points ${\left\{y_{i}\right\}}_{i=1}^{MN},$ which come from a union of *l* affine subspaces ${\left\{S_{i}\right\}}_{i=1}^{l}$, *l* is the parameter denoting the cluster number.

Step1. Sample *p* representative data **X** = [*x*_1_,*x*_2_,…*x*_*p*_] randomly form data points ${\left\{y_{i}\right\}}_{i=1}^{MN}$, the *k* nearest neighbors of each *x*_*i*_ is found.

Step 2. With (5) and (6), get the low dimensional representation of **X**, denoted by $X^{\prime }=[x_{1}^{\prime },x_{2}^{\prime },\ldots x_{p}^{\prime }]$.

Step 3. Embedding **Y** to feature space with (7) and (8), get the new representation **Y**′.

Step 4. Perform k-means on **Y**′ to obtain the whole labels.

**Output:** A 2-D matrix which records the labels of the clustering result of the HSI.

## Experimental results and discussion

In this section, we conduct a set of experiments to further evaluate the effectiveness of proposed algorithms for HSI. And considering the fairness, KASP-ASSC and RASP-ASSC and LI-ASP were used as benchmarks, because all of these algorithms are belong to approximate clustering methods.

### HSI data sets

**Fig 1:**
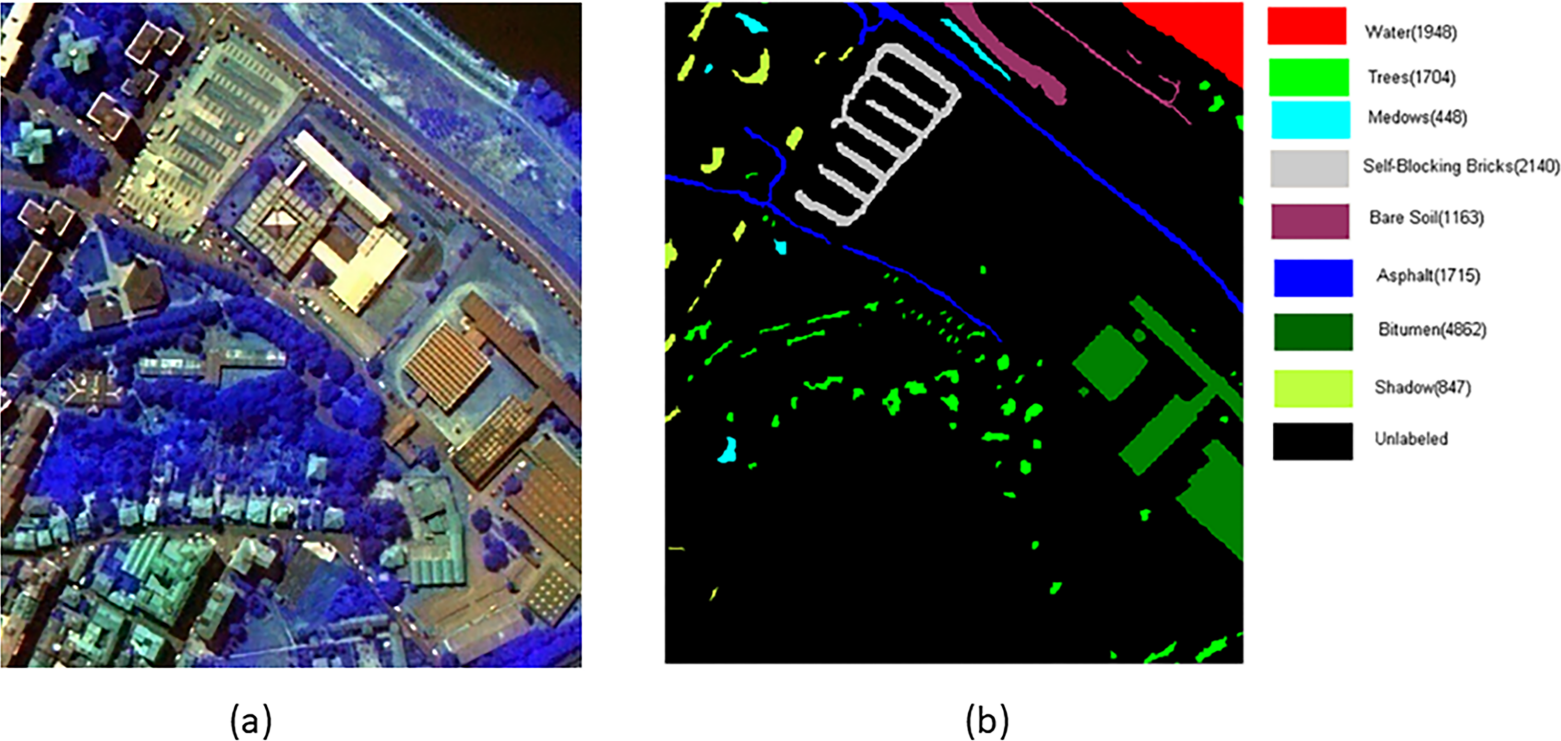
Part of pavia centre hyperspectral image. (a) The HSI in false color (RGB 3, 65,101), (b) Ground truth.

**Fig 2:**
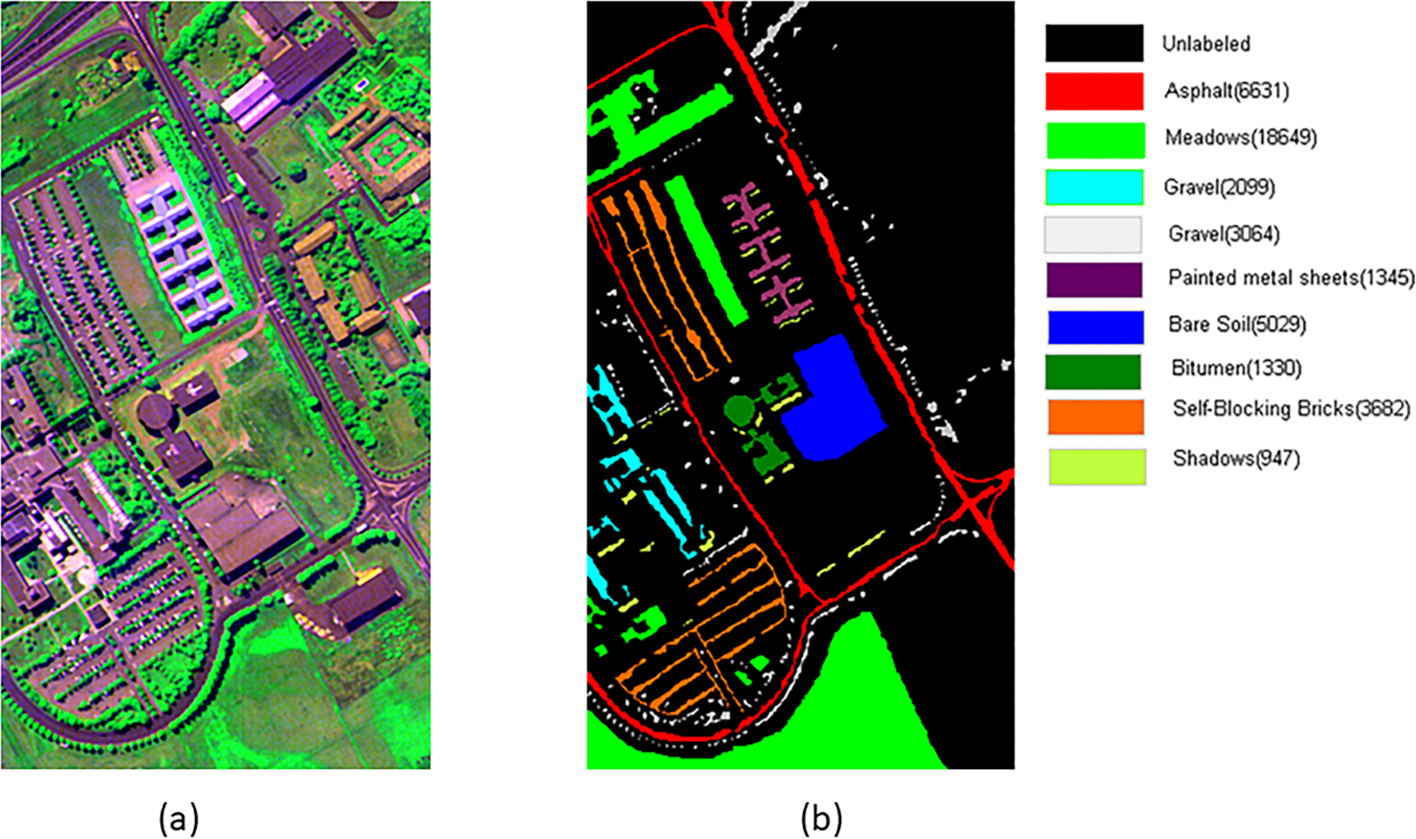
The paviaU hyperspectral remote sensing image. (a) The HSI in false color (RGB 64, 101,1), (b) Ground truth.

Two widely used HSI data sets are applied in our experiments, including PaviaU and Pavia Centre scenes [18, 19]. These are two scenes acquired by the ROSIS sensor during a flight campaign over Pavia, northern Italy. The number of spectral bands is 102 for Pavia Centre and 103 for PaviaU. Pavia Centre has a 1096×1096 pixels image, and Pavia University has 610×340 pixels, but some of the samples in both images contain no information and have to be discarded before the analysis. The geometric resolution is 1.3 meters. Both image ground truths differentiate 9 classes, and the discarded samples are shown as abroad black strips (Fig 1(B), Fig 2(B)). We trimmed a typical part of the image as the test area from Pavia Centre data set with the size of 399 ×348, which contains 8 main land-cover classes. The HSI in false color and its corresponding ground truth are shown in Fig 1 (A) and (B) respectively. Fig 2(A) and 2(B) shows the false color and its corresponding ground truth of PaviaU.

### Experimental set-up

Two aforementioned hyperspectral data sets with different imaging environment settings were used to validate the performance of the proposed methods. The number of clusters was set as a manual input, and the parameters of each algorithm were manually adjusted to the optimum. Both the visual clustering results and quantitative evaluations are given for each experiment.

In order to obtain quantitative evaluations, we adopted six evaluating indicators to measure the quality of clustering results. They are accuracy (AC) and normalized mutual information(NMI) [25, 26], which is often used to test clustering algorithm effect; producer’s accuracy, user’s accuracy, overall accuracy (OA), and kappa coefficient [18] which is usually used to verify the HSIs classification precision.

### Parameter analysis

**Fig 3:**
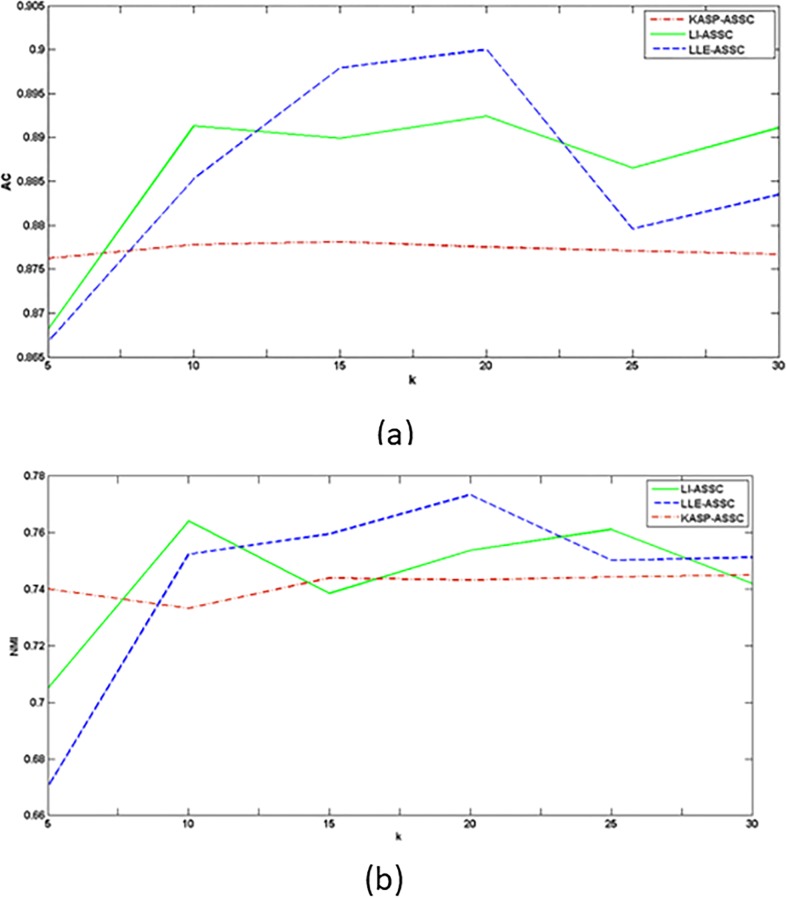
Analysis of parameter *k* (a) Change in the AC with various values of *k*. (b) Change in the NMI with various values of *k*.

In the course of extending the labels, the selected number of neighbor, *k*, has important impact on the clustering result. For guaranteeing both noise-immunity and detail-preserving for image based on local neighborhoods, Cai et.al [27] researched the number of neighbor in clustering algorithm. On this basis, we choose to compare the AC and NMI on paviaU set when *k* is changed from 5 to 30. Fig 3 shows the change in the AC and NMI of KASP-ASSC, LI-ASSC and LLE-ASSC corresponding to different *k* values, with the other parameters fixed.

It can be seen from Fig 3 that for LLE-ASSC and LI-ASSC, the precision changes with different values of *k*, which suggests that the neighbor structure plays a very important role in the clustering process. While for KASP-ASSC, the change is not so significant. Moreover, our proposed algorithms, no matter for AC or NMI, have obvious advantages compared with KASP-ASSC in general case. On the other hand, the time consumption will increase rapidly when *k* becoming bigger, but clustering effect is not be improved obviously. So in the following experiment, taking algorithm efficiency into consideration, we select manually adjusting the *k* value for each algorithm with it is less than 30 to get the effect that as good as it can.

**Fig 4:**
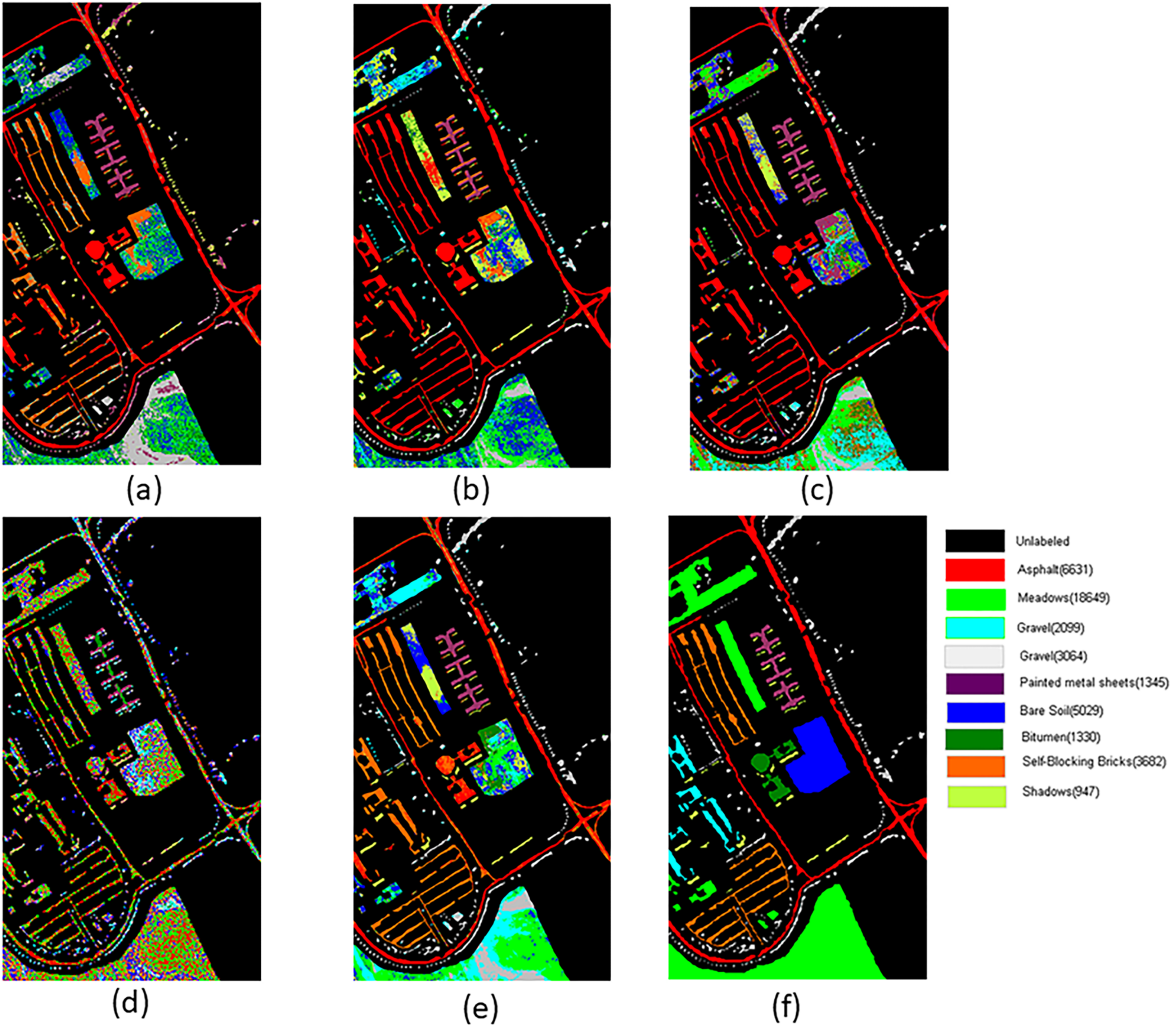
Cluster maps of the different methods with the PaviaU image(a)KASP-ASSC(k = 10) (b)RASP-ASSC(k = 10) (c)LI-ASSC(k = 10) (d)LI-ASP(k = 10) (e)LLE-ASSC(k = 20) (f)True Ground.

Fig 4 shows the visual clustering result. From Fig 4 it can be clearly observed that LI-ASSC and LLE-ASSC obtain more effective clustering results which contain less salt-and-pepper noise and significant less misclassification.

Next, we adopt the confusing matrix and the corresponding some common index to give the quantitative evaluation, the parameter *k* is same as showed in Fig 4. The result is listed in Table 1. In this table, the optimal value of each row is shown in bold, and the second best results are underlined. From Table 1, it can be seen that the clustering result of RASP-ASSC and KASP-ASSC are very poor and contains significant amounts of misclassifications, particularly for the Self-Blocking Bricks and Shadows class. While LI-ASSC and LLE-ASSC improves the clustering accuracy to a large degree by making use of the spatial neighborhood information in the course of extending labels. For the Self-Blocking Bricks class, the misclassification is significantly reduced by LI-ASSC which achieves a higher precision of 56.73%. Also KASP-ASSC obtains the better effect for Meadows, but the superiority is not so apparently. The OA and Kappa of LI-ASP are all very low, while LLE-ASSC has the best overall clustering effect.

**Table 1:** Quantitative evaluation of the different clustering algorithms for paviaU image.

Evaluation	Producer’s Accuracy (%)					User’s Accuracy (%)				
	RASP- ASSC	KASP- ASSC	LI- ASSC	LLE- ASSC	LI- ASP	RASP- ASSC	KASP- ASSC	LI- ASSC	LLE- ASSC	LI- ASP
Asphalt	44.78	44.87	73.74	**77.86**	29.23	**97.75**	97.68	82.22	79.19	33.93
Meadows	89.97	**89.98**	86.41	81.82	54.44	30.35	30.23	31.54	37.86	34.22
Gravel	0	0	**0.01**	0	2.36	0	0	0.04	0	2.1
Trees	63.29	63.5	**96**	54.24	27.71	73.11	73.65	59.6	**93.51**	30.74
Painted metal sheets	95.17	94.31	30.66	**98.65**	51.6	79.03	78.81	82.8	**98.14**	55.02
Bare Soil	22.34	22.51	24.78	**32.21**	15.26	25.55	**25.87**	25.51	25.13	19.15
Bitumen	0	0	0	0	0	0	0	0	0	0
Self-Blocking Bricks	0.2	19.69	**56.73**	49.65	7.5	0.05	6.41	0.06	**92.91**	19.58
Shadows	14.11	14.65	95.91	30.26	100	80.46	84.16	96.52	89.97	28.72
OA(%)	40.9	31.32	49.29	**51.51**	28.78
Kappa(%)	30.58	31.19	38.69	**42.54**	11.71

### Experiment on the pavia centre

**Fig 5:**
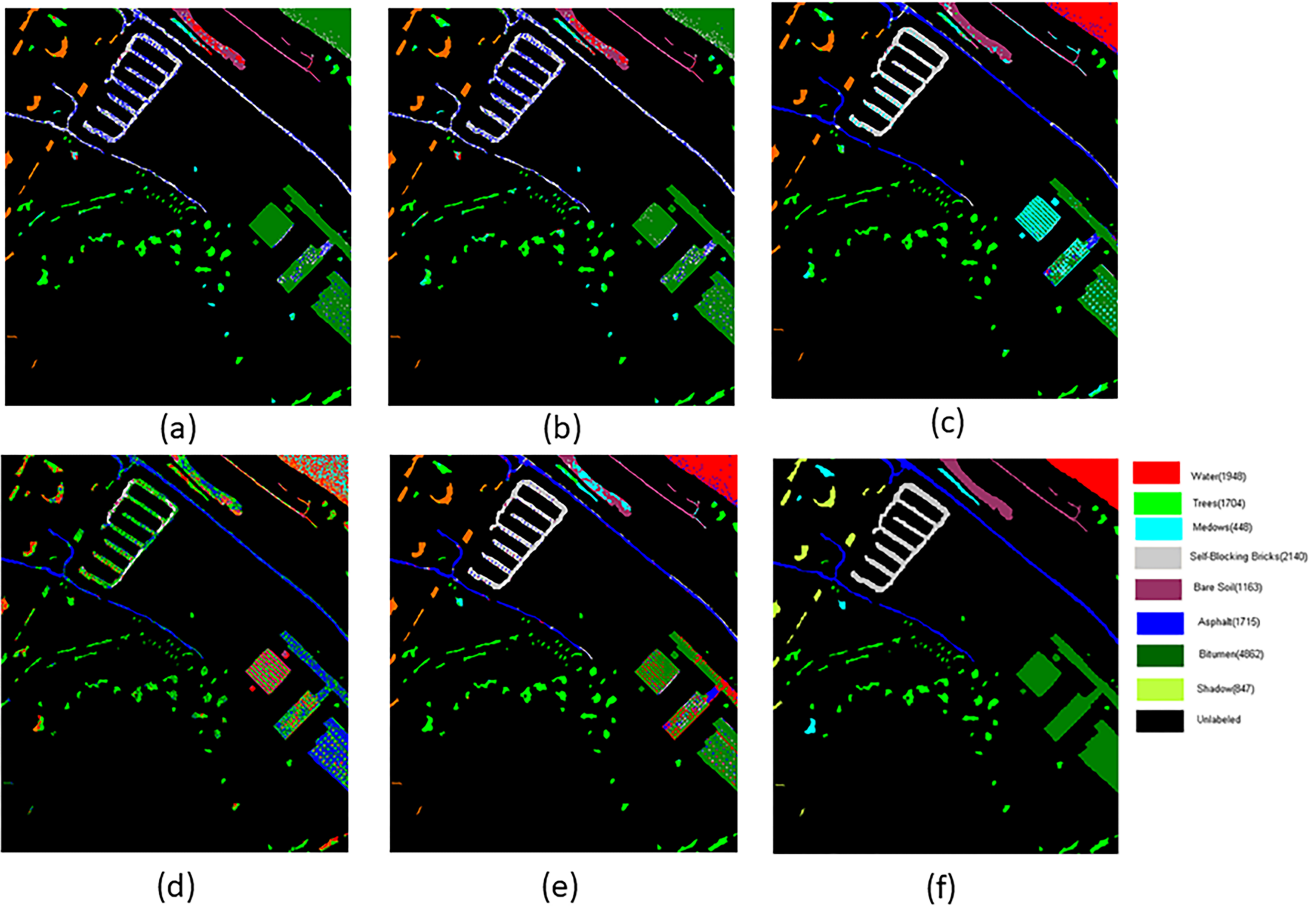
Cluster maps of the different methods with the Pavia centre image(a)KASP-ASSC(k = 20) (b) RASP-ASSC(k = 20) (c) LI-ASSC(k = 20) (d) LI-ASP(k = 10) (e) LLE-ASSC(k = 15) (f)True Ground.

In this experiment, for each algorithm, we chose the parameter k manually to obtain the best clustering effect. Other parameters involved in SSC adopted the default value. The clustering visual result is showed in Fig 5.

From Fig 5, we find that our algorithms have obvious advantage no matter from the visual and the quantitative effects. The water area is misclassified severely by LI-ASP,KASP-ASSC and RASP-ASSC. But our methods, LI-ASSC and LLE-ASSC, overcome this problem. In addition, the best clustering effect of self-blocking brick class is also obtained by LLE-ASSC.

The quantitative result of this experiment is listed in Table 2. Equally, Our algorithms obtain the more effective results. Especially for the water class, LI-ASSC gets the best result which precision achieves a higher producer’s precision of 98.51%. And for the Self-Blocking Bricks and Bitumen class, the more effective result is acquired by LLE-ASSC. And the OA and Kappa of KASP-ASSC is still very low, while LLE-ASSC have the best overall clustering effect.

**Table 2:** Quantitative evaluation of the different clustering algorithms for pavia centre image.

Evaluation	Producer’s Accuracy (%)					User’s Accuracy (%)				
	RASP- ASSC	KASP- ASSC	LI- ASSC	LLE- ASSC	LI- ASP	RASP- ASSC	KASP- ASSC	LI- ASSC	LLE- ASSC	LI- ASP
Water	0	0	**98.51**	70.61	57.5	0	0	94.82	94.61	45.43
Trees	**87.55**	86.43	86.04	84.06	0	88.73	91.2	97.3	**99.65**	0
Meadows	**43.41**	43.92	7.61	28.29	0	**35.27**	29.02	31.7	29.24	0
Self-Blocking Bricks	50.48	52.32	84.72	**86.54**	29.67	67.01	66.26	91.45	**92.52**	64.16
Bare soil	95.2	**96.66**	93.77	95.68	44.25	71.63	72.23	**82.8**	70.42	25.8
Asphalt	42.91	42.21	**83.19**	76.42	36.78	37.20	37.61	87.17	88.8	84.31
Bitumen	70.16	69.75	99.72	**99.76**	43.38	91.48	**92.29**	65.3	76.9	25.22
Shadow	99.76	99.64	**99.88**	99.53	100	99.17	99.29	99.17	**99.76**	24.79
OA(%)	66.52	66.87	81.45	**84.83**	36.69
Kappa(%)	57.91	58.27	78.16	**81.76**	24.5
Time(s)	1570.58	1485.32	1543.63	2138.26	1205.35

On the other hand, in this experiment, we compared the time consumption of these four algorithms, LI-ASSC has the faster convergence speed than LLE-ASSC, which speed is a little slower than KASP-ASSC. Theoretically, the computational complexity of LI-ASSC and LLE-ASSC is respectively O(*lkn*+2n+*τn*), O(*lkn*+3n+*τn*), and the computational complexity of KASP and RASP is about O(*lkn*). Where *l* represents the number of affine subspaces, *n* is the number of data samples, *k* is the neighbor number and *τ* is the number of iterations. From the order of magnitude, the difference of computational complexity is not so big. However, because the hyperspectral images have huge samples, parameter *n* has the most important influence on complexity. With the increasing of n, the computational time of LLE-ASSC increase rapidly.

To illustrate the robustness of the algorithm, we perform experiment 10 times on pavia centre image with same parameters. The average results are shown in Table 3. The value in blanket means square error. The conclusion is just similar with previous results. Our methods are more effective than KASP-ASSC and KASP-ASSC.

**Table 3:** The AC (std) and NMI (std) of clustering results on pavia centre image.

	KASP-ASSC	RASP-ASSC	LI-ASSC	LLE-ASSC	LI-ASP
AC	0.9581(0.0036)	0.9413(0.0039)	0.9635(0.0042)	0.9759(0.0091)	0.9324(0.0048)
NMI	0.8383(0.0140)	0.8405(0.0136)	0.8640(0.0115)	0.8968(0.0216)	0.7629(0.0185)

### Experiment on statistical significance

From these experiments mentioned above, we can find the proposed solution exhibited in general better performances than comparison methods. But on the same moment, we also noticed that in some cases these differences are very small. So, it is necessary to research the statistical significance.

Here, we also utilize the nonparametric McNemar test to evaluate the statistical significance in accuracy improvement with different algorithms. The McNemar’s test statistic for different algorithms can be calculated as [28]:

$$z=\frac{f_{12}-f_{21}}{\sqrt{f_{12}+f_{21}}}$$ (9)

Where *f*12 denotes the number of samples misclassified by algorithm 2 but not 1; and *f*21 means the number of samples misclassified by algorithm 1 but not 2. |*z*| is the absolute value of *z*. For 5% level of significance, the |*z*| value is 1.96. If a |*z*| value is greater than this quantity, the two classification algorithms have significant discrepancy.

Then we perform experiments for twenty times on both data sets by selecting representative samples randomly. For different size of two dataset, we chose 0.02% and 0.05% representative samples from paviaU and pavia centre respectively. Then, we perform the statistical analysis for the methods, LI-ASSC with RASP-ASSC, LLE-SSC with KASP-ASSC, and LLE-SSC with K-means, which can be tabulated as Table 4 and Table 5.

**Table 4:** Z values in the McNemar’s test result on paviaU image. And the 5% level of significance is selected.

PaviaU Image			
	LLE-ASSC & KASP-ASSC	LLE-SSC & K-means	LI-ASSC & RASP-ASSC
|z|	39.75	4.85	21.20

**Table 5:** Z values in the McNemar’s test result on pavia centre image. And the 5% level of significance is selected.

Pavia centre Image			
	LLE-ASSC & KASP-ASSC	LLE-SSC & K-means	LI-ASSC & RASP-ASSC
|z|	69.50	58.61	63.32

## Conclusion

In this paper, in view of hyperspectral image’s huge data size, we have introduced the approximate SSC algorithm to HSIs by treating each kind of land-cover class as a subspace based on approximate SC. According to our literature research, our algorithm and [18] are the only researches on the subject which apply SSC to HSIs classification. Reference [18] focused on improving the adjacent matrix and sparse model by utilizing of spatial-spectral information. But their work must be restricted by the memory capacity of computer. So the image is clipped to a very small size in their experiments [18]. For example, the PaviaU data set is cut to 200×100. But, yet the size in our experiment is 610×340. When the algorithm in [18] is used to deal with so big scale data set, the computational load will be very heavy.

In our works, we focus on how to improve the approximation performance. Faced with the shortcomings of KASP-ASSC, which directly use traditional extending label method in approximate course, we have proposed two novel ASSC based algorithms, i.e., LI-ASSC and LLE-ASSC, for HSIs. We take the local information into consideration in the ASSC model to promote the performance of the algorithm. The extensive experimental results, compared with some conventional approximate method, clearly verify that the proposed two ASSC based algorithms achieve a superior clustering performance and are competitive algorithms.

However, the proposed algorithms still have space for improvement. For instance, the problem of determining the parameter *k* adaptively is needed to be solved. And the representative data is selected randomly in our algorithms. This may be lack of uniformity in each class, which will be addressed in our future work. On the other hand, inspired by many excellent incremental algorithms [29], we also plan to design a similar incremental framework based on SSC.
